# Water as Green Solvent: Methods of Solubilisation and Extraction of Natural Products—Past, Present and Future Solutions

**DOI:** 10.3390/ph15121507

**Published:** 2022-12-03

**Authors:** Léo Lajoie, Anne-Sylvie Fabiano-Tixier, Farid Chemat

**Affiliations:** Groupe de Recherche en Eco-Extraction de Produits Naturel, Institut National de Recherche pour l’Agriculture, l’Alimentation et l’Environnement, Unité Mixte de Recherche 408, Département Agrosciences, Faculté de Chimie, Avignon Université, F-84000 Avignon, France

**Keywords:** green extraction, water solvents, water extraction, water solubilisation, natural products, sustainable development goals

## Abstract

Water is considered the greenest solvent. Nonetheless, the water solubility of natural products is still an incredibly challenging issue. Indeed, it is nearly impossible to solubilize or to extract many natural products properly using solely water due to their low solubility in this solvent. To address this issue, researchers have tried for decades to tune water properties to enhance its solvent potential in order to be able to solubilise or extract low-water solubility compounds. A few methods involving the use of solubilisers were described in the early 2000s. Since then, and particularly in recent years, additional methods have been described as useful to ensure the effective green extraction but also solubilisation of natural products using water as a solvent. Notably, combinations of these green methods unlock even higher extraction performances. This review aims to present, compare and analyse all promising methods and their relevant combinations to extract natural products from bioresources with water as solvent enhanced by green solubilisers and/or processes.

## 1. Introduction

**Figure 1:**
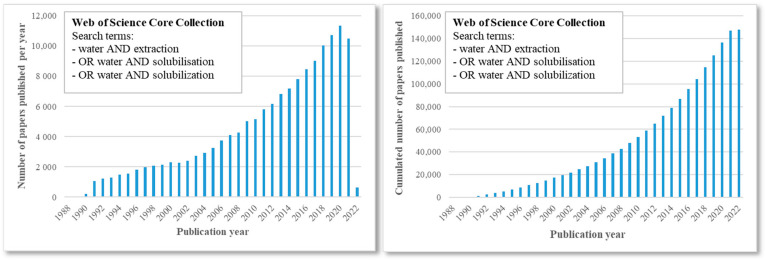
Search results depicting the total number of publications published per year (**left**) and the cumulated number by year (**right**) related to solubilisation and/or extraction using water as a solvent. Database: Web of Science Core Collection, time range: 1985–2022.

Water is seen as the solvent of life. Indeed, it is essential for every known living organism, and it may even be necessary for every unknown life form in the universe [1]. Within organisms, water acts as a useful solvent, supporting many vital physiological functions. Amongst other things, this solvent is able to solubilise numerous different molecules, it is part of various fundamental metabolic pathways and enables acid-base neutrality and enzyme function. All these advantages can also be utilised in the laboratory by chemists. Besides being a useful solvent, many researchers consider water as the greenest solvent in chemistry both from an experimental and an industrial point of view [2,3,4,5]. In addition, it is clear that there has been a continuous growth in interest for water in solubilisation and extraction since the 1980s, as shown in Figure 1.

Such an interest in using water as a solvent may be attributed to its easy accessibility and low cost, along with its green properties (non-toxicity, renewability, safety and ease of handling, ease of treatment and degradation, etc.).

Nevertheless, when water is used to solubilise or to extract natural products (NPs) from actual biological resources, this solvent appears to be relatively inefficient. For instance, the flavonoid rutin—which is theoretically quite polar according to its partition coefficient (Kow ≈ −0.47) [6]—is only sparingly soluble in water (S ≈ 130–150 mg/L) [6,7]. To overcome such a low efficiency in solubilising or extracting NPs, researchers have developed different methods to enhance the water solvent potential while taking advantage of its green qualities. By the turn of the millennium, Yalkowsky [8] had summed up the main methods to enhance the water solvent potential primarily in order to solubilise drugs more efficiently. These methods consist of the following: pH range and salts, cosolvents, surfactants, complexing ligands, inclusion complexes, stacking complexes and hydrotropes.

Once these initial seven methods to solubilise NPs in water were described at the beginning of the 2000s, additional ways to enhance the solubility of such compounds in water were introduced. Namely, switchable solvents were discovered in 2010 [9] followed by the Natural Deep Eutectic Solvents (NADES) first described in 2011 [10]. These two relevant solubilisation methods can easily be applied to modify water’s solvent properties. In fact, switchable solvents include switchable water and NADES can be dissolved in water so that the system remains an aqueous solvent. These techniques have continued to capture scientists’ attention to the extent that still today numerous articles are published with a view to extending their use and discovering and patenting new ingredients.

At this point, a total of nine methods have been established and are largely described as solubilisation techniques. All these methods may also be applied to the extraction of NPs from biological material. Whilst a relevant solubilisation technique does not necessarily constitute an efficient extraction technique—as was notably shown with the rosemary case study [11]—with the appropriate adjustments implemented, such techniques may be used for the purpose of both solubilisation and the extraction of NPs.

If we now consider water-based extraction methods, we can enrich this set of methods with four other innovative techniques, namely the use of enzymes [12], reactive extraction, in situ plant water extraction (ISPWE) [13] and subcritical water extraction (SWE) [14], which gives a total of thirteen water-based extraction methods.

This review introduces the basic principle of thirteen methods to enhance the solvent power of water in the green extraction of NPs. These methods are then analysed, compared and evaluated, with a score reflecting their green extraction global efficiency. Moreover, the future of water-based green extraction is expected to consist of relevant combinations of these methods to achieve higher extraction yields and greater extraction profiles. The interest of some of these combined methods will be discussed. Finally, the potential impact of these methods as a prospect is assessed through the prism of green extraction and the principles of green chemistry, then linked to the corresponding Sustainable Development Goals (SDGs) as defined by the United Nations.

## 2. Relevant Methods to Enhance the Solvent Potential of Water

### 2.1. Method Overview

**Figure 2:**
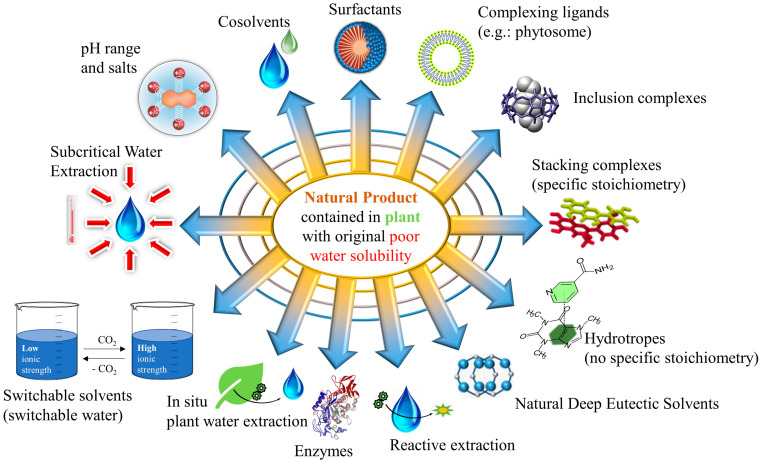
Present (from 2000 to 2020): most relevant water extraction methods for natural products (thirteen methods).

A total of thirteen methods are described in this section and illustrated in Figure 2.

Some of these methods rely on the addition of a chemical agent (organic, inorganic, biochemical) including pH range and salts, cosolvents, surfactants, complexing ligands, inclusion complexes, stacking complexes, hydrotropes, NADES and enzymes. Other methods are based both on the addition of a chemical agent and a physical treatment, such as reactive extraction and switchable solvents, whereas the two remaining methods involve the suitable physical treatment of the water, namely ISPWE and SWE.

Apart from reactive extraction, the use of enzymes, ISPWE and SWE, which are dedicated to the extraction of NPs, the rest of the methods can easily be applied to the solubilisation of NPs as well.

#### 2.1.1. pH Range and Salts

The use of salts or pH adjustment is largely implemented together with other methods. Hereinafter, we describe the specific effects of these two methods to enhance the solvent potential of water.

pH control is of major importance in understanding and monitoring water solubility and the extractability of multiple natural products. As can be seen in the case of anthocyanin delphinidin, the flavylium cation—which is dominant at a pH lower than 5—is the most soluble form of delphinidin and reaches a solubility of 71 mg/L in acidic water [15]. Therefore, a suitable way to extract such a compound from a plant resource such as berries is to use acidic water (e.g., pH = 2.3) to increase anthocyanin solubility and diffusion through the matrix [16]. pH adjustment may be performed with acids (e.g., hydrochloric acid) and bases (e.g., sodium or potassium hydroxide), as well as with salts (e.g., sodium carbonate to increase the pH or ammonium chloride to reduce it).

Salts can not only alter the pH but also monitor other water solvent properties. When added to water, salts split into ions that modify water behaviour. In particular, specific ion effects act on the surface charge and tension, electrostatic interactions and charge density [17]. When applied to solubilisation or extraction, useful salts such as kosmotropic and in particular chaotropic ones can be employed to alter interactions between water and NPs as well as their organisation. The direct consequence of these changes may be the increased solubility of NPs. Indeed, when a chaotropic salt is added to water, it weakens the interactions between each water molecule and each NP, thereby strengthening water-NP interactions to facilitate their solubilisation. This is called the salting-in effect [18]. Salts can also destabilise biological structures such as oil bodies or membranes, which could lead to coalescence and the release of protected NPs [19]. Finally, the use of salts could assist the mass transfer of NPs from the biological matrix to the water solvent [17].

The use of salts is very common in pharmaceutical industries dealing with natural products, as it is a very affordable and simple method to apply even at a very large scale for extraction and purification purposes. Nevertheless, this method could raise sustainability and cost-effectiveness questions if the salts used in the process have to be removed from the final product. Indeed, such removal could imply additional costly and energy-consuming downstream processing steps such as membrane filtrations [20]. In an ideal situation, salts should remain in the final product not only to simplify the process but also to potentially stabilise the extracted NP or even to enhance its properties (compared to those of the free NP solubilised in water) [21].

#### 2.1.2. Cosolvents

The use of cosolvents has to be the most obvious and common way to tune water solvent properties for green extraction purposes due to its ease of implementation, which consists of mixing water with one or more miscible solvents. Ethanol is one particularly notable cosolvent used with water.

The addition of a cosolvent to water is predominantly in a bid to reduce its polarity so that it behaves more like a medium- or low-polarity solvent and can therefore solubilise more non-polar NPs. Indeed, it was shown as early as 1931 that the addition of 50% (*w/w*) of ethanol to water at a temperature of 40 °C can reduce its polarity almost twofold (according to the dielectric constant value of the corresponding solvent: ε ≈ 73 for pure water and ε ≈ 45 for 50% ethanol) [22]. Apart from modifying water polarity, the addition of a cosolvent can also influence various parameters including, although not limited to, surface and interfacial tensions, viscosity, proticity and the ability to precipitate or crystallise a given NP [23]. Cosolvents could be added to water such as ethanol, polyethylene glycol, propylene glycol, glycerol and dimethyl isosorbide among others. In any case, the optimal ratio of cosolvent added to water should be studied with care. Indeed, it is unlikely that the best ratio to solubilise any given NP corresponds to the best ratio for its extraction. This noteworthy tendency was clearly demonstrated in the case of rosmarinic acid solubilisation and extraction from rosemary using aqueous ethanol [11]. The authors substantiated how pure ethanol was the best option to solubilise rosmarinic acid whereas the best ratio of ethanol to use in order to extract this NP was 30% (*V/V*).

Pharmaceutical industries are heavy users of cosolvents. For sure, this method is employed a lot for both extraction and purification steps. Before starting the process development and in particular its upscaling, the cosolvent has to be chosen wisely considering many parameters, including cost, availability, toxicity, efficacy and processing ability, recyclability and of course the amount added to water [24]. Once the appropriate cosolvent and its proportion are chosen, this method is quite easy to implement on an industrial scale. The environmental impacts of the corresponding process strongly depend on the target NP and the matrix extracted, as well as the nature of the retained cosolvent and its amount and also the galenic desired for the final product. As a matter of fact, if the cosolvent could remain in the final product, the process would be all the more sustainable.

#### 2.1.3. Surfactants

The use of surfactants is becoming a well-renowned technique in solubilisation and extraction of NPs. It involves the addition of surfactants, which are amphiphilic organic molecules of variable size, to water. Surfactants are surface-active molecules capable of forming micelles once their concentration is high enough (i.e., when C > critical micellar concentration—CMC). Micelles are colloidal-form clusters composed of surfactant molecules oriented in a way that separates hydrophobic moieties from water and exposes hydrophilic moieties to water [25]. Each surfactant has a given CMC.

The surfactant principle of action is to reduce surface tension. Different shapes of micelles are described: (classic) micelles, cylindric, layers and reverse [26]. These objects are able to solubilise non-polar NPs within their core, thus making those NPs more soluble in water (within micelles). The obtention and stabilisation of micelles are ensured by means of hydrophobic and hydration forces, π–π stacking interactions (in the case of aromatic-ring-containing surfactants) and hydrogen bonding [27,28]. Some examples of commonly used surfactants for NP solubilisation or extraction include non-ionic surfactants such as Triton X-100, Tween 20 or Tween 80, anionic surfactants such as docusate, cationic surfactants such as trimethyltetradecylammonium bromide, or even zwitterionic surfactants such as lecithin (glycerophospholipid mixtures) [8]. The extraction step must be performed using a concentration of surfactants higher than the CMC (typically around 1 to 10 mM) so that micelles are obtained and are able to solubilise the NPs within their core. Another useful property of micelles to exploit in green extraction processes is their cloud point. This consists of a temperature above which micelles are disorganised and therefore no longer water-soluble, which leads to dephasing. To purify the NPs after the extraction step, the user should bring these elements up to cloud point temperature and then add a centrifugation step to concentrate both the NPs and surfactants in the upper layer [26]. This extraction technique is called Cloud Point Extraction (CPE).

The study of surfactant benefits in pharmaceuticals is nothing new [8], though they seemed to be less frequently industrially applied than salts or cosolvents. Surfactants have shown to be clinically effective in emulsion when used in the oily phase [29], but their use in water for pharmaceutical purposes would still be uncommon. Nevertheless, promising results were obtained with NPs of growing importance in pharmaceuticals [30]. This method should not be a problem to scale up considering the generally low concentrations of surfactants involved, which also favours the sustainability of the corresponding process.

#### 2.1.4. Complexing Ligands

Contrary to the previous methods, which are quite common in studies of solubilisation and extraction of NP, the use of complexing ligands is still rather unusual in this field. This method consists of adding a complexing agent readily soluble in water to create a complex with the target compound [8]. The target compounds are typically metallic ions rather than NPs per se, which is why this method is uncommon in green extraction. One particular case of complexing ligands dissolved in water would be phytosomes. Phytosomes are phyto-phospholipid complexes composed of NPs and phospholipids [31]. They have a particle shape of variable diameter ranging from 50 nm up to 100 µm on average. Once complexed, NPs are far more soluble in water.

A complexing ligand typically contains two or more electron-donor groups. Complexation occurs as a result of ionic or covalent bonding. Once complexed, the compounds of interest become much more soluble in water and the obtained complex may serve as a drug delivery system [32]. Notable complexing ligands include ethylenediaminetetraacetic acid (EDTA), ethylenediamine disuccinate, or even citric acid [33,34]. Another unexpected complexing ligand recently reported was the NP dihydromyricetin itself, as detailed below [35].

The only relevant pharmaceutical application of this method would be the use of phytosome made with NP, therefore readily water-soluble. One frequently cited industrial producer of such active pharmaceutical ingredients (API) is Indena [36]. Notably, they claim the clinical efficiency of their Quercefit^®^ product (which is a phytosome made of quercetin) for reducing COVID-19 symptoms [37]. As this particular case of complexing ligand is quite new, it is still difficult to forecast the economic viability or sustainability of this technique in general.

#### 2.1.5. Inclusion Complexes

The use of inclusion complexes in the solubilisation and extraction of NPs has increased notably since 2010. In this method, an inclusion ligand is added to water. Inclusion ligands are amphiphilic molecules composed of a hydrophilic outer surface, which interacts with water and leads to its solubilisation, and an inner hydrophobic cavity able to host a hydrophobic moiety or an entire molecule [38]. An inclusion complex containing an NP will greatly increase its solubility in water. For instance, hesperetin formed as a complex with 2-hydroxypropyl-beta-cyclodextrin (HP-β-CD) is 400 times more soluble in water compared to its free form [39].

Such complexes are not only able to significantly improve NP solubility but also their stability and bioavailability. These complexes are obtained through several weak interactions such as ion–dipole, dipole–dipole, Van der Waals, electrostatic, hydrophobic and hydrogen bonds [39]. The most common inclusion ligands used in green extraction are cyclodextrins (CD). Various cyclodextrins are obtained from the enzymatic degradation of amylose, namely α-, β- and γ-CD (composed of 6, 7 and 8 glucose units, respectively) [38]. Additional CD have been developed from this ‘native CD’ basis through chemical modifications (e.g., HP-β-CD ‘modified CD’). Each CD has specific physicochemical properties, which is why the choice of CD should be decided rationally. The molar ratio of host-guest inclusion varies greatly from one host to another as well as from one guest to another (e.g., 1:1, 1:2, 2:1, 2:2, etc.). Large amounts of CD are generally required to solubilise NPs. For example, in the case of a 1:1 complexing molar ratio, 1 kg of CD is needed to solubilise 1 M of NP.

Inclusion complexes and in particular CDs were extensively used by many pharmaceutical companies in a wide variety of treatments since the 1970s [40]. More or less one hundred pharmaceutical products involving CDs were approved up to now, according to a supplier of these inclusion agents [41]. From a practical industrial point of view, processes based on the use of inclusion complexes are simple to implement, whatever the production scale. Additionally, the corresponding method is globally sustainable as it does not imply additional treatment steps. Moreover, these bio-based solubilisers will remain on the final product to improve their bioavailability and are initially introduced by simple stirring in water at room temperature [42].

#### 2.1.6. Stacking Complexes

Stacking complexes have barely been used in extraction, instead constituting a solubilisation technique [8]. Nevertheless, this could serve as a powerful method to extract NPs in a sustainable way that could favour the extract’s bioactivity. A stacking agent is generally a small, organic and amphiphilic molecule, with at least a decent water solubility. These agents are able to create aggregates (different from micelles) with hydrophobic molecules. The obtained stacking complexes are therefore much more soluble in water than the isolated hydrophobic compound ever was. Stacking is the major stabilising force involved in these complexes. More precisely, π–π interactions occur and enable the stacking phenomenon. Such complexes appear with a given molar ratio. The ideal target NPs for this method are π-electron donors such as compounds containing double bonds. Different geometric configurations of π–π stacking interactions have been described: edge-to-face stacking, offset stacking and face-to-face stacking. Given the weakness of their stabilising interactions, stacking complexes consist of drug delivery systems with huge potential [43].

Stacking complexes are naturally occurring in vivo systems in many bioresources, especially in plant cell walls. Indeed, these complexes are of major importance in plant cell wall organisation and stabilisation. For instance, polysaccharides such as pectin and phenolic compounds such as anthocyanidins rely on these interactions [44]. In addition, stacking complexes are responsible for the coloured appearance of many flower pigments, which are NPs of interest such as anthocyanidins [45]. This is why this method could be relevant for implementation in green extraction, using actual NPs as stacking agents.

These complexes have been studied for pharmaceutical purposes as a way to enhance the water solubility of existing and recognised APIs since the 1950s. Although, the π–π stacking interactions involved in the solubility enhancement of the studied drugs started to be well described and understood in the 1970s [46]. As mentioned above, stacking complexes appear to be theoretically suitable drug-delivery systems, nonetheless, they are still at the research stage, and it seems that there is still no real industrial use for this method. This is mostly due to safety concerns about the complexing agents used for pharmaceutical goals [43]. If we now consider the perspectives for industrial developments, we could forecast that the corresponding process would not be that difficult to implement. Indeed, no extra energy-consuming steps would be involved, especially if the agent were to be kept in the final products.

#### 2.1.7. Hydrotropes

Hydrotropes are small, amphiphilic surface-active molecules similar to stacking agents. However, contrary to stacking complexes, this method has been increasingly used in green extraction since 2000. A critical concentration similar to the CMC (established for surfactants) has been defined for hydrotropes and is known as the Minimum Hydrotropic Concentration (MHC). Above the MHC, hydrotropes form aggregates and begin to efficiently solubilise the target NP. A common order of magnitude for this MHC is 1 M. Even so, it is still unclear whether this aggregate occurs solely in the presence of the hydrophobic NP or with the hydrotrope alone [47].

The underlying physicochemical principles of this method are the same as those of stacking complexes. Nevertheless, the only significant difference with hydrotropes is that they can aggregate without any specific stoichiometry [8]. Many hydrotropes have been used successfully in green extraction, including sodium salicylate and sodium cumene sulfonate. Hydrotropes also exhibit a cloud point, which is useful to exploit in green extraction; this technique is called hydrotropic extraction. In short, hydrotropes are introduced into the water at a concentration higher than the MHC (typically exceeding 1 M) before starting the extraction process. Once the target NPs are extracted, the temperature is increased above the hydrotropic cloud point, which makes it easier to recover the NPs through phase separation with centrifugation for instance.

The application of hydrotropes in the pharmaceutical field is still limited to the research stage and it seems that there is still no approved drug involving such solubiliser, as in the case of stacking agents. Yet, it is suggested that the use of hydrotropes would be suitable for the extraction of NPs at an industrial scale. As a matter of fact, some hydrotropes with high-temperature stability appear to be easily reusable, thus giving way to a promising and sustainable method [48]. In the case of hydrotropes which could not be removed from the final product, the main concern about their use in the pharmaceutical field is their high concentration which is needed to reach satisfying extraction yields. This concentration range typically raises problems of toxicity and thus limits the potential applications of this method [49].

#### 2.1.8. NADES

Natural deep eutectic solvents (NADES) are fully organised liquids composed of naturally occurring metabolites found in most living cells [50]. They were introduced in 2011 and were then quickly and increasingly applied in green extraction [51]. Whilst water sometimes forms an intrinsic part of NADES as an actual component, it can also be used to dissolve initially dry NADES to reduce their viscosity for instance. In this sense, we can consider NADES as being an aqueous solvent whereby water represents at least half of the total composition. NADES are obtained by mixing different compounds at very specific molar concentrations.

The molecular structure and stability of NADES are based on the extensive hydrogen bonding that occurs between compounds. There are five categories of NADES according to their composition: acid and base, neutral, neutral with acids, neutral with bases, and amino acids containing NADES. Most NADES exhibit very low toxicity and are sometimes even edible [50].

Aqueous NADES are extremely well suited to use in green extraction both in a laboratory and on an industrial scale. As a matter of fact, their components are remarkably low-cost, NADES are relatively easy to formulate, and they are non-toxic and highly biodegradable. Amongst the most renowned NADES, we can count choline chloride:urea, choline chloride:lactic acid, choline chloride:ethylene glycol, glucose:fructose:sucrose, and malic acid:glucose [52]. Each of these NADES could easily be dissolved in at least 50% water to make it an aqueous solvent with practical advantages such as viscosity and cost reductions.

Regarding applications in the health industry, certain compositions of aqueous NADES were patented by Givaudan in 2015 [53]. This method was used by the company to produce at least two cosmetic active ingredients [54]. In 2017, another company named Gattefossé launched its cosmetic active ingredient made from an aqueous NADES composed of glycerin and fructose [55]. If we now consider strictly speaking the pharmaceutical industrial application of this method, it might not exist yet. Nevertheless, as this method was successively used in a related health industrial sector, it seems feasible to apply it in pharmaceutical processes. In fact, the corresponding process is globally sustainable even if NADES tend to be viscous. If viscosity is a problem for given applications, the water dilution can be simply increased. Again, to remain sustainable, the process should not include NADES removal. This purification is hard to achieve because of many NADES properties including their generally very low vapor pressure [51].

#### 2.1.9. Reactive Extraction

Reactive extraction is a novel concept and we first coined the term in this review. It covers the extraction methods involving the chemical modification of the target NP(s) by means of a reactive extractant. After the reaction, the NP becomes much more water-soluble. The NP may be transformed back into its initial form in the final extract or could also remain chemically modified, depending on the applied process. Very few papers describe this kind of transformation and of course, they do so without employing the expression *reactive extraction*. Most of the reactive extractants we have identified are either salts [56] or surfactants [57].

Reactive extraction is based on the use of chaotropic salts, surfactants, or even pH adjustments, as well as physical modification such as a change in temperature or a processing step inducing phase separation. All these techniques can lead to one or more modifications of the target NP to make it more water-soluble so that extraction yields are increased.

This method features several advantages for green extraction. For instance, it could help in reducing the number of unitary operations while using natural molecules such as choline hydroxide as a reactive extractant [57].

Since this method is very new, there is logically no pharmaceutical industrial application to date. Nonetheless, NP of pharmaceutical interest could be extracted thanks to reactive extraction, such as for example hesperidin [56]. The chemicals or process involved are cheap and simple to implement. In addition, these operations will not induce extra energy consumption, thus enabling the green process. Overall, this method looks promising for future developments but still suffers from a lack of scientific data and conceptualisation.

#### 2.1.10. Enzymes

Enzymes are natural active proteins (macromolecules of a least roughly 50 amino acids) synthetised by organisms and which act as biocatalysts. These active proteins are highly specific and can perform the same reactions millions of times at an exceedingly high pace (high turnover rate). The water extraction of NPs may be greatly increased by the use of enzymes [26].

Hydrolases and lyases are the most useful enzyme categories in green extraction. Indeed, they catalyse the hydrolysis and other kinds of bond cleavage of many molecules such as structural polysaccharides, proteins and lignin, thus enabling the disassembly of plant cell walls [58]. Such plant cell wall degradation may lead to easier access of NPs for water solvents. Moreover, the degradation itself can release phenolic compounds from the cell wall [59].

Most of the enzymes used in green extraction are obtained from fungus and bacteria strains. The following enzymes have been successfully used for NP extraction from plant sources: cellulases, pectinesterase, polygalacturonase, rhamnogalacturonan hydrolase, alpha-amylase, peptidase, trypsin, papain and more [59]. Typically, one or more of these enzyme categories are added into water and the plant matrix is extracted through stirred maceration, with pH and temperature adjustments to fit enzyme needs. Even more complicated NPs to extract such as some polyunsaturated fatty acids can be isolated using this method by adding a cold-pressing step to the process [60].

No application was identified in pharmaceuticals using enzymes during the extraction step. Nevertheless, there were many promising lab-scale results related to the extraction of NP as explained above. Furthermore, active ingredients for dietary supplements and cosmetics are currently produced by at least one company. Indeed, Biolie which is specialised in enzymatic extraction already launched three active ingredients for dietary supplements and more or less a dozen for cosmetics using this method [61]. Even if this method is not the easiest to optimise nor to scale up, it would be possible to produce pharmaceuticals using enzymes which are efficient green biocatalysts.

#### 2.1.11. ISPWE

In situ plant water extraction involves physical treatments capable of extracting plant water in situ, along with NPs. This extraction method does not require the addition of any solvents, as the water contained in the plant is sufficient. The two main technologies required to achieve this solvent-free extraction were identified as microwaves and Pulsed Electric Fields (PEF). The mass transfer of NPs from the wet matrix to the exterior is greatly enhanced by these treatments.

In the case of Solvent-Free Microwave Extraction (SFME), both heat and mass transfers occur in the same direction so that NPs are efficiently extracted by water in situ. Contrary to most processes that involve conventional heating (i.e., surface heating), microwaves ensure that selective volume heating is applied to the matrix (from its core to the exterior) [61]. As for PEF, which is a non-thermal process, the in situ water extraction relies on the irreversible electroporation of the matrix cells. This kind of extensive cell membrane or wall destruction happens if the potential difference between the two electrodes is high enough (E >> E_critical_) [62].

In green extraction, this method could efficiently be applied to any matrix with a high-water content (wet sample) for it to serve as a solvent. Such physical treatments are exceptionally fast and typically last just a few minutes. These processes are also low energy-consuming [63].

To the best of our knowledge, there was no industrial application of ISPWE for pharmaceutical purposes yet. We could forecast that the use of SFME or PEF for pharmaceutical production is quite far from being widely adopted. Indeed, these simple treatments lead to raw complex extracts, which will then require many purification steps involving other technologies to reach levels of purity matching pharmaceutical needs. ISPWE is more suitable for the obtention of raw products as close as possible to the material of origin.

#### 2.1.12. Switchable Solvents

Switchable solvents are green solvents that are readily tuneable in a reversible way using the appropriate trigger, for instance, through reactions with CO_2_. Other acids such as hydrochloric acid could serve as a trigger, but generally, CO_2_ is preferred because of its advantages (ease of handling related to its gaseous state, generally non-toxic, available and inexpensive). Amongst the developed switchable solvents, switchable water appears to be an excellent water-based solubilisation and extraction method to target non-polar NPs. Switchable water is obtained by adding a base soluble into the water, such as N,N,N’,N’-tetramethylbutane-1,4-diamine [64]. This base enables ‘the switch,’ which consists of the addition or depletion of CO_2_ to monitor the ionic strength of the aqueous solution. At the end of the process, it is possible to remove the base from the water solution to make it clean and safe once again.

Bases such as amines or polyamines may be used to obtain switchable water. In the absence of CO_2_, the aqueous solution has a low ionic strength because of the amine. If CO_2_ is added to the solution, the base is protonated once or more depending on its protonatable sites, giving the aqueous solvent a sudden, strong ionic strength [65]. In a typical green extraction protocol involving switchable water, a base is introduced into the water before adding the matrix containing the NPs. Depending on the solubility of the target compounds, that switchable water is either carbonated or uncarbonated. CO_2_ bubbling can be used to achieve the classic 1 atm loading capacity of interest for switchable water. Depending on the process, once the NPs have been extracted, CO_2_ pressure may be adjusted to monitor their solubility. Finally, the base can easily be recovered from the water after the complete removal of CO_2_.

It would appear that there is currently no industrial application of switchable water in the pharmaceutical field. This method is still at a research level, nonetheless, promising results of NPs extraction were obtained thanks to it. In particular, it was shown that the base involved in obtaining ‘the switch’ is highly recyclable at the end of lab-scale processes [66]. This suggests that the corresponding potential industrial process could be sustainable.

#### 2.1.13. SWE

Subcritical water extraction is a particular physical state of water approaching its critical point. More precisely, SWE is typically obtained and used at a temperature between 100 to 200 °C and maintained at a pressure of up to 22.1 MPa, enabling it to stay in liquid form rather than becoming a gas [67]. Compared to water used at an ambient temperature and pressure, SWE is a less polar solvent that behaves more like methanol and is, therefore, able to solubilise and extract less polar NPs that ambient water could not.

In terms of physicochemical properties, SWE shows a reduced dielectric constant approaching those of acetonitrile or methanol. Its viscosity and surface tension are also decreased, which enables a deeper penetration of the liquid into the matrix used for extraction. Finally, the density and the diffusion rate of SWE are lower than in the case of ambient water, which leads to an enhanced mass transfer of the dissolved ions and molecules [68].

SWE is generally far more efficient and convenient than extraction with water at ambient temperatures or hot water without pressure control, especially because of the speed of the corresponding process [59]. Moreover, after the extraction step, SWE once again becomes ambient water and the non-polar compounds may spontaneously form a separate phase in which they are easy to recover since the compounds that were once soluble in SWE are no more.

There has not been an application of SWE in pharmaceuticals to date. Nevertheless, this technique is actually used by at least two major industries to produce active ingredients for the nutraceutical and cosmetic markets. These are Lubrizol [69] and Sensient [70,71]. The corresponding extracts are rich in bioactive NPs and have complex compositions. That is why we can hypothesise that it rather meets the needs of nutraceutical and cosmetics industries, more than pharmaceutical ones which are generally looking for high-purity molecules. In any case, SWE appears to be a green and sustainable technique efficient for the extraction of NP and is already mature enough for some markets of the health industry.

### 2.2. Successful Cases of Green Extraction Using Each Method

Each method described in the previous subsection has been applied to the green extraction of NPs multiple times and revealed interesting results. Successful cases illustrating the advantage of each method are detailed in Table 1.

Now that every method has been briefly explained, the following section will focus on analysing them. Each method will also be compared and rated to help the reader choose between all possibilities depending on their needs.

### 2.3. Method Analysis, Comparison and Rating

In Table 2, these methods are described and then compared in terms of relevant criteria for both academic researchers and people in the industry. These criteria consist of the investment cost to implement the method, the ease of the corresponding operation, the hydropathy of target NPs and the processing time, as well as the main pros and cons.

**Figure 3:**
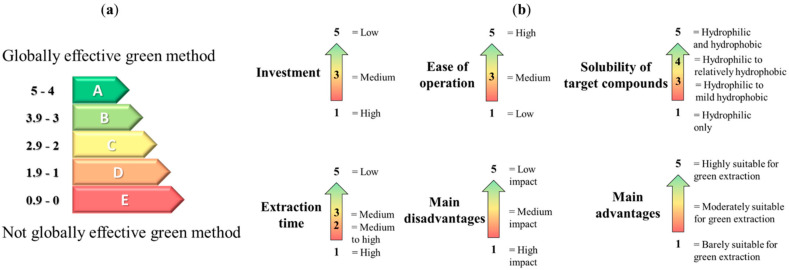
Caption of the rating index used for the different methods analysed (**a**) and for every criterion of each method (**b**).

The rating captions and details are shown in Figure 3 and each criterion rating is depicted in Table 3.

According to this analysis, hydrotropes and NADES are the most globally efficient methods to enhance the solvent potential of water (grade A). The use of hydrotropes is quite easy, very low-cost, and fast, which makes it a cost-effective method to implement globally. As for NADES, their naturality, low cost and ease of dissolubility in water explain their top grade. Nine other methods were given an average grade of B. Finally, the use of enzymes and SWE was given the worst grade (C). In the case of SWE, this grade is attributed to the extremely high investment costs (mainly in the industry) as well as the partial thermal degradation of the target NPs and other products. With regard to the use of enzymes, the difficulty of operation and high extraction time resulted in this C grade. Although both these methods remain relevant to tune water solvent potential, they should be thoroughly studied and optimised for the desired applications. Of course, whilst this overall result could help the reader in choosing the appropriate method, important adjustments and the compliance of the method with target NPs are fundamental criteria to maximise extraction yields.

## 3. Future of Water-Based Extraction: Combined Methods at 2 or 3 Levels

**Figure 4:**
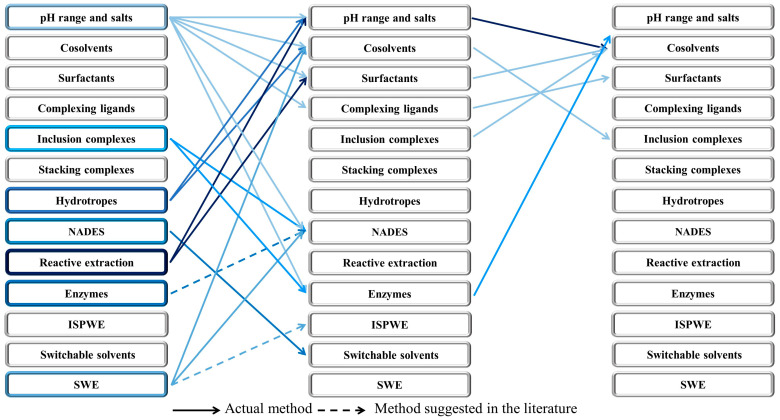
Future: Combinations of water extraction techniques to maximise natural compound recovery. Classic arrows indicate actual method found in the literature, while arrows with broken lines represent methods suggested by authors.

Combinations of methods are useful for two main reasons. Firstly, combinations can lead to an increase in the solubility and extractability of the target NPs. Secondly, combinations can also reduce the intrinsic toxicity of a chemical agent used in a single method, by reducing its concentration. Some agents from one method may be combined with another agent from the same method, which is the case for pH range and salts, cosolvents, surfactants, inclusion complexes, stacking complexes and hydrotropes. Other chemicals are particularly well suited for combining with another method, as is the case with pH range and salts, inclusion complexes, stacking complexes and hydrotropes [8]. In Figure 4, we report useful examples of combinations found in the literature to reach outstanding extraction yields.

As an outstanding first case study of a two-method combination, the reader should consider Sed et al.’s publication [80]. Here, the team working with Prof. Jessop (who discovered switchable solvents) combined the use of NADES with a switchable solvent. The extraction yields of protein, carbohydrates and lipids obtained from microalgae were excellent while using almost only natural ingredients. According to the author, this was the most benign switchable solvent system ever developed.

Chen et al.’s relevant proof of concept involving the combination of three methods is also relevant for further reading [81]. In this article, the authors adjusted the pH using salts in addition to the use of a sugar-based cosolvent before combining it with the well-known surfactant Span 20 to efficiently extract the oil contained in shelled walnuts. With these water modifications, the oil recovery yield exceeded 90%.

We strongly believe that these sorts of multi-method combinations could be the future of the water-based green extraction of natural products for two main reasons; firstly, because of the higher yields potentially obtained and also because of the reduced toxicity induced by the excipients thereby introduced in lower quantities compared to their use in single methods.

## 4. Contribution of These Methods in Terms of Sustainability: Consolidating the SDGs

**Figure 5:**
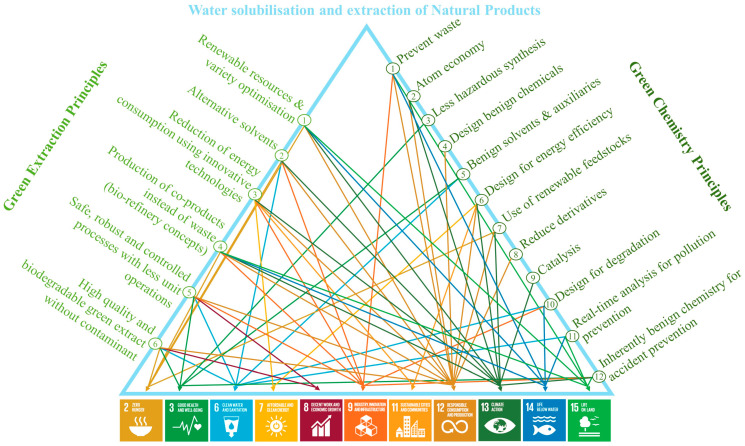
Contribution to the SDGs of the principles of green extraction and green chemistry related to the use of water as a solvent for solubilisation and the extraction of natural products. SDG 2: Zero Hunger; SDG 3 Good Health and Well-being; SDG 6: Clean Water and Sanitation; SDG 7: Affordable and Clean Energy; SDG 8: Decent Work and Economic Growth; SDG 9: Industry, Innovation and Infrastructure; SDG 11: Sustainable Cities and Communities; SDG 12: Responsible Consumption and Production; SDG 13: Climate Action; SDG 14: Life Below Water; SDG 15: Life on Land.

The principles of green chemistry defined by Anastas, Warner and Zimmerman have raised significant attention since their introduction, notably because they represent a precise way to tackle climate change [82,83]. The principles of green extraction introduced by Chemat et al. [84] are strongly related to these principles of green chemistry, although they are much more adapted to the extraction of natural products. In this respect, the principles of green extraction are relevant for addressing global challenges and threats as well. Indeed, the use of water for solubilisation and the extraction of natural products not only meets the vast majority of both the principles of green chemistry and green extraction but also fits with 11 out of the 17 SDGs. These correlations are depicted in Figure 5.

Here, we provide some practical examples to consolidate numerous SDGs through the use of water in the green extraction context, whether in a single laboratory or on an industrial scale. (SDG 2) Zero Hunger: water is a very low-cost and easily accessible solvent that enables food production through the extraction of nutritional compounds from bioresources; (SDG 3) Good Health and Well-being: replacing toxic and harmful solvents with water is a good idea to protect both the extract producer and the final product consumers; (SDG 6) Clean Water and Sanitation: some of the methods described here could be used to extract pollutant from water and thereby recover fresh water that is safe to drink and to use for domestic purposes; (SDG 7) Affordable and Clean Energy: the majority of these techniques require very low energy input, meaning that the overall demand could be reduced; (SDG 8) Decent Work and Economic Growth: water-based processes could guarantee safe work free from exposure to harmful chemicals (thereby replaced) for employees in a production facility and such processes could also facilitate economic growth, as they do not require the acquisition of expansive products or technology; (SDG 9) Industry, Innovation and Infrastructure: water-based processes could be innovative and lead to new, clean label products; (SDG 11) Sustainable Cities and Communities: no harmful chemicals are involved in water-based processes so the effluents do not harm nearby towns or cities; (SDG 12) Responsible Consumption and Production: water-based processing is an obvious way to ensure the clean production of environmentally friendly products; (SDG 13) Climate Action: reducing the use of harmful chemicals is a relevant way to limit greenhouse gas emissions and their overall impact; (SDG 14) Life Below Water: wastewater derived from water-based processes will not be difficult to treat and will limit the corresponding aquatic contamination, (SGD 15) Life on Land: the reduction in greenhouse gas emissions induced by the use of water-based processes protects life on land.

## 5. Conclusions

In this review, we first introduced the seven traditional solubilisation methods for using water as the primary solvent. Next, we presented a more in-depth description of the thirteen most relevant and popular water-based solubilisation and NP extraction methods. We also presented the eventual application(s) of each method in pharmaceutical industries. These methods were then analysed and compared. As a result, it could be easy to choose one or a few methods to prioritise for their desired application. In this way, the use of hydrotropes and NADES should be considered as turnkey solutions to tune the solvent potential of water. Subsequently, we presented the advantages of combining some of these methods, which can lead to a reduction in the solvent’s intrinsic toxicity while unlocking higher extraction yields. We forecast that these combinations of methods will be increasingly adopted by both academic and industrial researchers because of their ever-growing potential. We then postulated that these methods could help in meeting global challenges; more precisely, having a positive impact on 11 out of the 17 SDGs.

## Figures and Tables

**Table 1:** Presentation of a successful case of green extraction for each method.

Method	Matrix	Target NP(s)	Experimental Conditions	Results (Compared to the Control)	Refs.
pH and salts 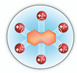	Bitter almond (*Prunus amygdalus* L.) kernel powder 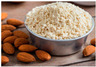	Monounsaturated and polyunsaturated fatty acids contained in the oil (e.g., oleic and linolenic acids) 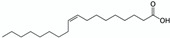 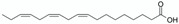	1 h stirred maceration at 84 °C with sodium bicarbonate (0.4 M) in water Matrix/Solvent = 1/5 *w/w* 30 min centrifugation at 2368× *g* Control: same treatment with pure water	91% oil extraction yield with sodium bicarbonate (>55% with control) Reduced creamy phase Less toxic compounds extracted (hydrocyanic acid)	[19]
Cosolvents 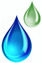	Propolis powder 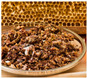	Phenolic compounds (e.g., vanillin) 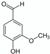	5 h maceration at room temperature with 20% propylene glycol in water Matrix/Solvent = 1/10 *w/V* Control: same treatment with pure water	20% propylene glycol in water can double the extraction of total phenolic compounds from propolis and triple the antioxidant activity	[72]
Surfactants 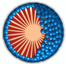	Cannabis (*Cannabis sativa* L.) resin 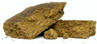	Δ9-tetrahydrocannabinol (THC) 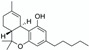	CPE with non-ionic surfactant Dowfax 20B102 (ethylene oxide-propylene oxide copolymer-based) 4 h maceration at 45 °C Matrix/Solvent = 1/2000 *w/V* Controls: same treatment with pure hexane or pure methanol	Dowfax is approximately 4 times more efficient than hexane and methanol controls (62% THC extraction versus 14 and 17%, respectively) Possibility to enhance this extraction yield by combining this method with the use of a salt: 81% THC recovery with the addition of 1% Na_2_SO_4_	[30]
Complexing ligands 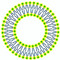	Chinese vine tea (*Ampelopsis grossedentata*) leaf powder 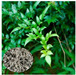	Dihydromyricetin 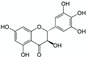	Reverse method to extract NP from a matrix: use of the complexing properties of the NP itself (chelation extraction). 2 h maceration at 90 °C (pH = 2, HCl 2M) with zinc salt (complexation of ionic zinc by dihydromyricetin). Reverse extraction of dihydromyricetin with disodium EDTA (stronger binding affinity with zinc than dihydromyricetin). Dihydromyricetin released in high quantities in the extract and EDTA-zinc complexes easily removed via filtration. Matrix/Solvent = 1/20 *w/V*	1 2% dihydromyricetin recovery yield and 94% purity (versus 8 and 91%, respectively obtained with the reference method of repeated crystallisation [54])	[35]
Inclusion complexes 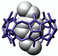	Giant knotweed (*Polygonum cuspidatum*) root powder 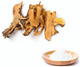	Resveratrol 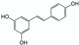	1 h ultrasound-assisted extraction with 1.5 wt% β-CD in water Matrix/Solvent = 1/50 *w/V* Conventional solvent: same treatment with pure methanol Control: same treatment with water	125 times more resveratrol extracted with 1.5 wt% β-CD than with the control. As efficient as pure methanol. Similar antioxidant activities between methanolic and β-CD aqueous extracts	[73]
Stacking complexes 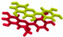	*Sennae folium* (dried *Cassia angustifolia* Vahl or *Cassia acutifolia* Delile leaf) powder 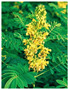	Sennoside A 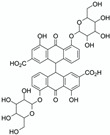	30 min ultrasound-assisted extraction 0.1% NaHCO_3_ Matrix/Solvent = 1/16.67 *w/V* Precipitation extraction with berberine (added in excess) Filtration, washing and drying Controls: organic bases such as sulphanilamide or ammonium thiosulfate	More efficient selective sennoside extraction with berberine than with controls. Specific binding molar ratio: 2:1 (berberine:sennoside A)	[74]
Hydrotropes 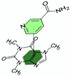	Poison Devil’s pepper (*Rauwolfia vomitoria*) root bark powder 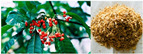	Reserpine 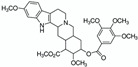	40 min stirred maceration at room temperature with hydrotrope sodium cumene sulfonate dissolved in water (2 M). Matrix/Solvent = 1/10 *w/V* Control: same treatment with pure methanol Reference (assumed total extraction): 48 h reflux extraction (Soxhlet) with chloroform	Hydrotrope extraction as efficient as the reference Soxhlet extraction with chloroform, 72 times shorter. Also more than 3 times greater than methanol.	[75]
NADES 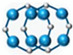	Powder from traditional Chinese medicine herbal preparation JinQi Jiangtang (consisting of *Coptis chinensis*, *Astragalus membranaceus*, and *Lonicera japonica*) 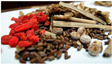	Phenolic compounds and alkaloids (e.g., chlorogenic acid and groenlandicine) 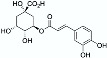 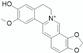	1 h ultrasound-assisted extraction with aqueous NADES (50% water content) composed of choline chloride:Levulinic acid 1:2 (mol:mol) Matrix/Solvent = 1/125 *w/V* Controls: same treatment with 70% methanol and with water	Aqueous NADES more efficient than both controls in extracting chlorogenic acid and groenlandicine	[76]
Reactive extraction 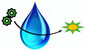	Italian blood orange (*Citrus sinensis*) peel (industrial by-product) 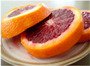	Hesperidin 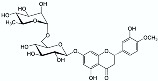	1 h stirred maceration at 60 °C after pH adjustment using calcium hydroxide (pH = 12), followed by neutralisation with hydrochloric acid (pH = 6) to modify hesperidin structure before resin purification. Matrix/Solvent = 1/5 *w/V*	40% extraction yield, 93% purity	[56]
Enzymes 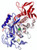	Syrah grape (*Vitis vinifera* ‘Syrah’) pomace 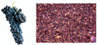	Phenolic compounds (e.g., *p*-coumaric acid) 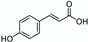	1 h stirred maceration (orbital shaker, 125 rpm) at 45 °C, pH = 5 (acetate buffer at 50 mM) using cellulase and tannase alone and in combination. Control: same treatment with water	Individually, cellulase and tannase greatly enhanced extraction yields of gallic acid, p-coumaric acid, and total phenolic compounds (from 2 to 8 times) compared to control. Combination of both enzymes categories is beneficial.	[77]
ISPWE 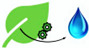	Lettuce (*Lettuce sativa*) 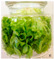	Phenolic compounds (e.g., quercetin) 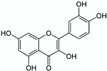	20 min SFME at 250–300 W (lab and pilot scale, 4 and 150 L reactors respectively) Conventional extraction: 5 min ultra-homogenisation (4000 rpm) at room temperature with 80% ethanol (Matrix/Solvent = 1/10 *w/V*)	Quercetin and luteolin at least 5 times more concentrated in SFME extracts (lab and pilot scales) compared to conventional extracts.	[78]
Switchable solvents 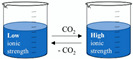	Pure compounds in water (solubilisation tests)	Various NPs (e.g., benzoic acid and capsaicin) 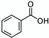 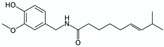	Switchable water obtained with N,N,N’,N’-tetramethylbutane-1,4-diamine (1:5 base:water V:V ≈ 0.9 M) with or without CO_2_ (1 atm of air or CO_2_) Control: pure water (1 atm of air)	Capsaicin and benzoic acid far more soluble in switchable water than in control (877 and 73 times respectively)	[64]
SWE 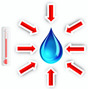	*Pseuderanthemum palatiferum* (Nees) Radlk. leaf powder 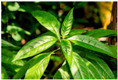	Phenolic compounds, flavonoids and saponins. 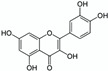	15 min SWE at 130 to 190 °C, 80 bar Matrix/Solvent = 1/70 *w/V* Conventional solvent and extraction procedures: 19 h stirred maceration with methanol at 25 °C (Matrix/Solvent = 1/100 *w/V*) 7 h Soxhlet reflux with 70% ethanol (Matrix/Solvent = 1/100 *w/V*) 30 min stirred maceration with hot water at 80 °C (Matrix/Solvent = 1/25 *w/V*)	SWE most efficient and fastest method SWE extracts far richer in NPs, exhibit 2 to 20 times more antioxidant activity, as well as more antimicrobial power (inhibition zone) compared to conventional extracts	[79]

**Table 2:** Present (from 2000 to 2020): Practical comparison and rating of these techniques.

Method	pH and Salts	Cosolvents	Surfactants	Complexing Ligands	Inclusion Complexes	Stacking Complexes	Hydrotropes	NADES	Reactive Extraction	Enzymes	ISPWE	Switchable Solvent	SWE
System description	Addition of salts to increase solubility	Addition of solvent(s) to tune water polarity, proticity and viscosity	Addition of surfactants to create micelles	Addition of a complexing agent to capture the target compound	Addition of an inclusion agent to host the target compound	Addition of a stacking agent to increase solubility	Addition of organic or natural agent to increase solubility	Addition of natural, small organic molecules at specific molar ratio	Addition of salts to extract and simultaneously transform the target compound	Addition of enzymes in water under specific conditions to denature the matrix	Physical treatments to extract plant metabolites using its own water content	Addition of organic bases and CO_2_ to switch water behaviour	Water at a high temperature and pressure to keep it in liquid state
Investment	Low	Low	Low to medium	Low	Medium	Medium to high	Low to medium	Low	Low	Medium	High	Low to medium	High
Ease of operation	High	High	Medium	Medium	High	Medium	High	Medium	Low to medium	Low	Medium to high	Medium	Medium
Hydropathy of target NPs	Hydrophilic and lipophilic	Hydrophilic to mildly lipophilic	Hydrophilic to mildly lipophilic	Hydrophilic and lipophilic (with phytosomes)	Hydrophilic to relatively lipophilic	Hydrophilic and lipophilic	Hydrophilic to mildly lipophilic	Hydrophilic to relatively lipophilic	Hydrophilic and lipophilic	Hydrophilic and lipophilic	Hydrophilic to mild lipophilic	Hydrophilic and lipophilic	Hydrophilic to mildly lipophilic
Extraction time	Medium	Medium to high	Medium	Medium to high	Medium	Medium	Low	Medium	Medium to high	High	Low	Medium to high	Medium
Main disadvantages	Very specific (precise conditions necessary)	Limited concentration authorised in food products, obligation to remove it	Surfactant removal	Lack of data in plant extraction	Difficult to combine with other methods	Lack of data in plant extraction	High concentration of hydrotropes needed	Patented use	Lack of data in plant extraction	Enzyme price, precise conditions necessary	Not particularly tuneable or easy to implement	Still needs toxic organic agents (albeit in small quantities)	Not suitable for thermosensitive molecules, high pressure
Main advantages	Useful method mainly if used in combination with others, intensification techniques	Cosolvents could be part of next steps in formulation	Simultaneous extraction of polar and apolar molecules	Extremely target-specific, potential drug delivery system (enhanced stability), could be part of next steps in formulation	Extremely target-specific, potential drug delivery system (enhanced stability), could be part of next steps in formulation	Extremely target-specific, could be part of next steps in formulation	Hydrotropes could be part of next steps in formulation	Biomimetic (natural, GRAS)tuneable quantity of water added, enables intensification	Highly efficient while using very low-cost agents	Matrix pretreatment	No solvent needed and short treatment	Ease of recovery of both product and extractant and specifically designed to facilitate industrial implementation	Tuneable solvent power
	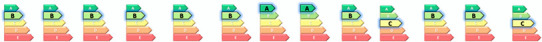												

Each method was given an average rating indicating the global efficiency, with A being the best rating and E the worst (the rating details are explained in Figure 3 and Table 3).

**Table 3:** Details about the rating given to every criterion of each method.

Method	pH and Salts	Cosolvents	Surfactants	Complexing Ligands	Inclusion Complexes	Stacking Complexes	Hydrotropes	NADES	Reactive Extraction	Enzymes	ISPWE	Switchable Solvent	SWE
Investment	5	5	4	5	3	2	4	5	5	3	1	4	1
Ease of operation	5	5	3	3	5	3	5	3	2	1	4	3	3
Solubility of the target compounds	5	3	3	5	4	5	4	4	5	5	3	5	3
Extraction time	3	2	3	2	3	3	5	3	2	1	5	2	3
Main disadvantages	2	2	2	3	3	3	2	4	3	2	2	2	2
Main advantages	2	3	4	4	5	3	4	5	5	4	5	5	4
Average score	3.7	3.3	3.2	3.7	3.8	3.2	4.0	4.0	3.7	2.7	3.3	3.5	2.7
Equivalent letter	B	B	B	B	B	B	A	A	B	C	B	B	C

## Data Availability

Data sharing not applicable.
